# The suppression of the differentiation of adipocytes with *Mallotus furetianus* is regulated through the posttranslational modifications of C/EBPβ

**DOI:** 10.1002/fsn3.3551

**Published:** 2023-07-05

**Authors:** Touko Nakano, Yutaro Sasaki, Toshio Norikura, Yusuke Hosokawa, Mayu Kasano, Isao Matsui‐Yuasa, Xuedan Huang, Yoshinori Kobayashi, Akiko Kojima‐Yuasa

**Affiliations:** ^1^ Department of Food and Human Health Sciences Graduate School of Human Life Science Osaka City University Osaka Japan; ^2^ Department of Nutrition Aomori University of Health and Welfare Aomori Japan; ^3^ Department of Nutrition Graduate School of Human Life and Ecology Osaka Metropolitan University Osaka Japan; ^4^ Department of Pharmacognosy School of Pharmacy Kitasato University Tokyo Japan

**Keywords:** antiobesity effect, C/EBPβ, in vitro experimental system using 3T3‐L1 preadipocytes, in vivo obesity model mice, *Mallotus furetianus* extract, posttranslational modification

## Abstract

Obesity is a major risk factor for various chronic diseases, especially lifestyle‐related diseases. Therefore, finding a protective substance against obesity and elucidating its molecular mechanism is one of the most important problems for improving human health. In this study, we investigated the antiobesity effect of *Mallotus furetianus* extract (MFE). The aim of the study was to examine the in vivo and in vitro effects of MFE on lipid synthesis. We examined the effect using an in vivo experimental system with obesity model mice and an in vitro experimental system with 3T3‐L1 preadipocytes. We found that the treatment of MFE significantly suppressed the increase in body weight and adipose tissue weight and morphological changes in the liver and adipose tissue of the obesity model mice. In the in vitro experimental system, we revealed that MFE treatment suppressed the expression of transcription factors such as C/EBPα, C/EBPβ, and PPARγ, which are involved in the early differentiation of 3T3‐L1 preadipocytes. As a result, the ability to synthesize triacylglycerol was suppressed. An interesting finding in this study was the clarification that MFE decreases the expression of C/EBPβ through post‐translation modifications (PTMs), followed by the transcriptional suppression of PPAR𝛾 and C/EBP𝛼.

## Graphical abstract

The treatment of *Mallotus furetianus* extract (MFE) significantly suppressed the increase in body weight and adipose tissue weight in obesity model mice. The treatment of MFE suppressed the expression of transcription factors that involves at the early stage of differentiation of 3T3‐L1 preadipocytes. The expression of C/EBPβ, one of the transcription factors, was decreased at the level of posttranslational modification by MFE, followed by the suppressions of PPARγ and C/EBPα transcriptionally.

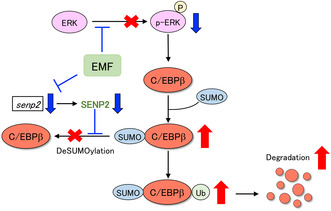

## 1 INTRODUCTION

Obesity is a major risk factor for various chronic diseases, especially lifestyle‐related diseases. Therefore, finding a protective substance against obesity and elucidating its molecular mechanism is one of the most important problems for improving human health.

Antiobesity effects induced by natural products include induction of apoptosis, cell cycle arrest or retardation, and interference with transcription factors or signaling pathways, which occur early in adipogenesis. Among them, transcription factors such as peroxisome proliferator‐activated receptor‐γ (PPAR γ) and CCAAT/enhancer‐binding protein (C/EBP) family play an important role in the differentiation into adipocytes (Gregoire et al., 1998). Expression of C/EBPβ is induced in the early stages of adipocyte differentiation. It has been reported that in mice in which C/EBPβ is knocked down, adipose tissue development is inhibited and fat weight is reduced (Tanaka et al., 1997), which also indicates that C/EBPβ plays an important role in adipocyte differentiation. However, the regulatory mechanism of the expression of C/EBPβ remains unclear. Zhang et al. reported that C/EBPβ was regulated transcriptionally during adipogenesis (Zhang et al., 2004). On the other hand, some researchers have shown that C/EBPβ protein expression is regulated by posttranslational modifications (Edward & Yeh, 2009; Fujita et al., 2021; Guo et al., 2015; Liu et al., 2013; Watanabe et al., 2020).

In recent years, food ingredients with antiobesity effect have been in the limelight. For example, resveratrol, a type of polyphenol compound contained in red wine, has been reported to show antiobesity effects by suppressing inflammation caused by tumor necrosis factor‐α in 3T3‐L1 preadipocytes (Ahn et al., 2007). 1′‐Acetoxychavicol acetate, a component obtained from *galangal* seeds and rhizomes of ginger, has antioxidant and anti‐inflammatory effects, and in studies using 3T3‐L1 preadipocytes and high‐fat diet rats, it has been shown to have an antiobesity effect (Ohnishi et al., 2012). In addition, various food ingredients such as catechin (Moon et al., 2007), curcumin (Ejaz et al., 2009), soy isoflavone (Ørgaarol & Jensen, 2008), and carob pods (Fujita et al., 2021) have been found to have antiobesity effects. However, the detailed mechanisms by which these components suppress the differentiation into adipocytes remain unclear.

*Mallotus furetianus* is a kind of tropical plant of the *Euphorbiaceae* family, which is native to Hainan Island, China, and has been traditionally used as a folk medicine for gallbladder disease (Liu et al., 2011). *Mallotus furetianus* leaf extract (MFE) has been reported to have antiarteriosclerosis (Liu et al., 2011) and antisteatosis effects (Huang et al., 2017). However, the antiobesity effect of MFE and its mechanism have not yet been clarified.

In this study, we investigated the effects of MFE using both in vivo and in vitro experimental systems. Animal experiments are required to confirm that MFE is absorbed from the intestine and reached target organs. Obesity is also closely related to the development of hepatic steatosis caused by the accumulation of excessive fat (Haslam & James, 2005), and excessive accumulation of fat in the liver causes inflammation and more severe liver disease (Inzaugarat et al., 2011). Therefore, it is important to examine with an experimental animal to investigate the effect on the liver in addition to the adipose tissue.

Therefore, the purpose of this study was to clarify the antiobesity effect of MFE by in vivo experimental system of mice and to elucidate the molecular mechanism of the expression of C/EBPβ by MFE in vitro experimental system using 3T3‐L1 preadipocytes. In this study, we found that MFE significantly suppressed the increase in body weight and adipose tissue weight in the in vivo obesity model of high‐fat diet mice. We have also shown that the treatment of MFE suppressed the expression of transcription factors that involves at the early stage of differentiation of 3T3‐L1 preadipocytes and reduced triglyceride (TG) accumulation.

## 2 MATERIALS AND METHODS

### 2.1 Preparation of MFE

The dried *Mallotus furetianus* leaves (Lot No. 20110424044) were purchased from Hainan Ecological Green Tea Limited in China. *Mallotus furetianus* leaves (400 g) were extracted three times with hot water (40 L). After filtration, the filtrate was evaporated in rotary evaporator at 42°C and lyophilized to dryness and got 100.7 g of MFE (Huang et al., 2017). Some of the components of *Mallotus furetianus* leaves have been reported (Chen et al., 2017; Huang et al., 2017; Li et al., 2022; Lin et al., 2006).

### 2.2 Animal experimentation

Thirty‐six 8‐week‐old C57BL/6J male mice were purchased from SLC Inc., and caged at a constant temperature (25°C) for 1 week. The light–dark cycle was 12 h, and diet and water were freely ingested. After preliminary feeding, animals were divided into four groups; (1) control diet (Control), (2) high‐fat diet (HF), (3) control + 0.24% MFE diet (C + MFE), and (4) HF + 0.24% MFE diet (HF + MFE), and bred for 32 days. The diet composition was prepared according to AIN‐93M (Table 1). Three mice were bred in one cage, and two cages were used per group. After the end of breeding, the mice were allowed to inhale 5% isoflurane (Pfizer) as anesthesia. Blood was collected from the inferior vena cava under anesthesia to euthanasia, and then the liver, adipose tissue, kidney, and spleen were removed. Organs were washed with saline (0.9% NaCl solution), weighed, and the liver was frozen in liquid nitrogen. Blood was centrifuged to obtain plasma. Plasma and liver were stored at −80°C until measurement. Animal experiments were conducted in accordance with the Osaka City University Animal Experiment Committee Regulations (Permit No. S0056).

**TABLE 1:** Diet composition.

	Control	HF	C + MFE	HF + MFE
Casein	140.000	140.000	140.000	140.000
Lard	0	310.000	0	310.000
L‐Cystine	1.800	1.800	1.800	1.800
Cornstarch	465.692	233.019	463.292	230.619
Alpha‐cornstarch	155.000	77.673	155.000	77.673
Sucrose	100.000	100.000	100.000	100.000
Soybean Oil	40.000	40.000	40.000	40.000
Cellulose powder	50.000	50.000	50.000	50.000
AIN‐93M mineral^a^	35.000	35.000	35.000	35.000
AIN‐93 vitamin^b^	10.000	10.000	10.000	10.000
Choline hydrogen tartrate	2.500	2.500	2.500	2.500
tert‐Butylhydroquinone	0.008	0.008	0.008	0.008
MFE	0	0	2.4	2.4
Total (g)	1000	1000	1000	1000

^a^ Composition in g/kg diet: calcium carbonate, anhydrous, 357.00; potassium phosphate, monobasic, 250.00; sodium chloride, 74.00; potassium sulfate, 46.60; potassium citrate, 28.00; magnesium oxide, 24.00; ferric citrate, 6.06; zinc carbonate, 1.65; manganous carbonate, 0.63; cupric carbonate, 0.324; potassium iodate, 0.01; sodium selenate, anhydrous, 0.01025; ammonium paramolybdate, 4 hydrates, 0.00795; sodium meta‐silicate, 9 hydrates, 1.45; chromium potassium sulfate, 12 hydrates, 0.275; lithium chloride, 0.0174; boric acid, 0.0815; sodium fluoride, 0.0635; nickel carbonate, 4 hydrates, 0.0306; ammonium vanadate, 0.0066; powdered sucrose, 209.7832.

^b^ Composition in g/kg diet: nicotinic acid, 3.000; calcium pantothenate, 1.600; pyridoxine‐HCl, 0.700; thiamin‐HCl, 0.600; riboflavin, 0.600; folic acid, 0.200; D‐biotin, 0.200; vitamin B_12_ (cyanocobalamin; 0.l% in mannitol), 2.500; vitamin E (all‐*rac‐*α‐tocopheryl acetate; 500 IU/g), 15.00; vitamin A (all‐*trans*‐retinyl palmitate; 500,000 IU/g), 0.800; vitamin D_3_ (cholecalciferol; 400,000 IU/g), 0.250; vitamin K (phylloquinone), 0.075; powdered sucrose, 974.655.

### 2.3 Histopathological analysis of liver and adipose tissue

Liver and retroabdominal adipose tissue samples were taken from each mouse, fixed with a 10% neutral buffered formalin solution, and the samples were embedded in paraffin blocks and sectioned at 5 μm thickness with sliding microtome (Leica Biosystems). The specimens were stained with hematoxylin–eosin (H&E) staining. In brief, the specimens were deparaffinized using xylene and hydrated by gradually decreasing the concentration of ethanol. The specimens were then stained with Mayer's hematoxylin solution for 4 min, followed by color development in running water. Furthermore, they were stained with Eosin solution for 4 min and dehydrated by gradually increasing the concentration of ethanol. After clearing the specimens with xylene, they were cover slipped using a mounting medium.

### 2.4 Cell culture

We used 3T3‐L1 mouse fibroblasts as an in vitro model for adipocyte differentiation (Green & Kehinde, 1975, 1976; Green & Meuth, 1974).

Mouse‐derived 3T3‐L1 preadipocytes (JCRB9014) were purchased from the National Institutes of Biomedical Innovation, Health and Nutrition JCRB Cell Bank. Differentiation into adipocytes was performed according to the previously reported method (Fujita et al., 2021). 3T3‐L1 precursor adipocytes were cultured in Dulbecco's modified Eagle's medium (DMEM) containing 10% fetal bovine serum (FBS) in a 5% CO_2_ incubator at 37°C. Two days after reaching confluence (day 0), adipocyte differentiation was induced in DMEM medium containing 10% FBS supplemented with 0.25 μM dexamethasone, 0.5 mM 3‐isobutyl‐1‐methylxanthine, and 0.2 μM insulin (DMI). Two days later, the cells were treated with DMEM‐containing 10% FBS supplemented with 0.2 μM insulin for another 2 days, and then cultured in DMEM‐containing 10% FBS. The differentiation was completed on the 8th day from the start of differentiation induction. MFE was dissolved in dimethyl sulfoxide (DMSO). The control was treated with DMSO only. The final DMSO concentration in the medium was less than 0.5%.

### 2.5 Cell viability

Cell viability was measured by neutral red assay (Riddell et al., 1986). After culturing, Neutral Red Reagent (Fujifilm Wako Pure Chemicals) was added to a final concentration of 50 μg/mL, and the cells were cultured at 37°C for 2 h. Then, the cells were washed with 2 mL of 1% formaldehyde/1% CaCl_2_ mixture, 1 mL of 1% CH_3_COOH/50% ethanol mixture was added, and the cells were allowed to stand at room temperature for 30 min. The absorbance of the extract was measured at a wavelength of 540 nm using a spectrophotometer (JASCO V‐730 BIO Spectrophotometer). Cell viability was calculated as (A540‐treated cells/A540 of appropriate control) × 100% after correcting for background absorbance.

### 2.6 Oil Red O staining

The intracellular triglyceride was measured by Oil Red O staining (Ramirez‐Zacarias et al., 1992). 3T3 L1 adipocytes were washed twice with 2 mL of Ca^++^‐ and Mg^++^‐free phosphate buffer saline (PBS (−)) and fixed with 60% ethanol. Then, 1 mL of Oil Red O solution was added, and the mixture was allowed to stand at room temperature for 20 min, and then washed once with 50% ethanol and twice with 1 mL of ultrapure water. Then, the staining solution was extracted with 1 mL of 2‐propanol, and the absorbance was measured at a wavelength of 520 nm using a spectrophotometer (JASCO V‐730 BIO Spectrophotometer).

### 2.7 Measurement of glycerol‐3‐phosphate dehydrogenase (GPDH) activity

3T3‐L1 adipocytes were collected 8 days after the start of differentiation. The cells were washed twice with 1 mL PBS (−). Cells were harvested with 350 μL triethanolamine/EDTA buffer using a cell scraper and crushed with a sonicator (BIO RUPTOR, COSMO BIO Co., LTD.). After centrifugation (13,000×*g*, 5 min, 4°C), the supernatant was used for enzyme assay, and the GPDH activity was measured according to the method of Weis and Green (Weis & Green, 1979). The enzyme activity was calculated using the extinction coefficient of nicotinamide adenine dinucleotide (NADH) of 6.22 mM^−1^ cm^−1^ and was calculated based on the change in NADH per 3 min. Enzyme activity was expressed as a value relative to the control (100%).

### 2.8 Quantitative reverse transcription‐polymerase chain reaction (qRT‐PCR)

Total RNA of 3T3‐L1 preadipocytes was extracted using High Pure RNA Isolation Kit (Roche). The quality and quantity of the extracted Total RNA were evaluated using a 2100 Bioanalyzer (Agilent Technology). The cDNA was synthesized using the PrimeScript RT Reagent Kit (TaKaRa Bio Inc.). qRT‐PCR was performed on the StepOnePlus PCR System (Thermo Fisher Scientific) using TB Green Premix Ex Taq II (TaKaRa Bio Inc.). The running program was as follows: 95°C for 30 s, followed by 40 cycles at 95°C for 5 s and 60°C for 30 s. The primer sequence is shown in Table 2, and the value normalized to β‐actin was used as the mRNA expression level. StepOne software v2.2.2 (Thermo Fisher Scientific) was used for delta–delta CT analysis.

**TABLE 2:** Primers used for real‐time PCR.

	Sense	Antisense	Accession number
*C/EBPβ*	5′‐ACGGGACTGACGCAACACA‐3′	5′‐TGCTCGAAACGGAAAAGGTTC‐3′	NM_001287738
*PPARγ*	5′‐GGAGCCTAAGTTTGAGTTTGCTGTG‐3′	5′‐TGCAGCAGGTTGTCTTGGATG‐3′	NM_001127330
C/EBPα	5′‐TTGAAGCACAATCGATCCATCC‐3′	5′‐GCACACTGCCATTGCACAAG‐3′	NM_001287514
*SENP2*	5′‐CCTGGTGGTGATGGACCTAAGA‐3′	5′‐GCTGAGGAATCTCATGTGGCTT‐3′	XM_030249324
*β‐Actin*	5′‐CATCCGTAAAGACCTCTATGCCAAC‐3′	5′‐ATGGAGCCACCGATCCACA‐3′	NM_007393

Abbreviation: PCR, polymerase chain reaction.

### 2.9 Western blotting

3T3‐L1 preadipocytes were collected in RIPA buffer with a cell scraper and sonicated using a sonicator (BIO RUPTOR, COSMO BIO Co, LTD.). RIPA buffer contains protease inhibitors (phenylmethylsulfonyl fluoride [PMSF], leupeptin, and pepstatin), phosphatase inhibitors (sodium fluoride), and tyrosine phosphatase inhibitors (sodium orthovanadate). After centrifugation (13,000×*g*, 10 min, 4°C), sample buffer was added to the supernatant, and then it was heated at 90°C for 5 min. Samples were kept at −80°C before use. The protein concentration was assayed using the Pierce™ BCA protein assay kit (Thermo Fisher Scientific). Total protein (20 μg) was separated by 10% SDS‐PAGE and then transferred to a polyvinylidene difluoride membrane (Merck Millipore). Then, blocking was performed with 3% bovine serum albumin for 1 h, and the membrane was incubated with the primary antibody at 4°C overnight. Anti‐C/EBPβ (H‐7; Santa Cruz Biotechnology), anti‐C/EBPα (14AA; Santa Cruz Biotechnology), anti‐PPAR (E‐8; Santa Cruz Biotechnology), and anti‐p44/42MAPK (Erk1/2; 137F5) XP® Rabbit mAb (Cell Signaling Technology) were diluted 1:1000. Anti‐Phospho‐p44/42MAPK (Erk1/2; Thr202/Tyr204; D13.14.4E) XP® rabbit mAb (Cell Signaling Technology) was diluted 1:2000. Furthermore, GAPDH (D16H11) XP rabbit mAb (Cell Signaling Technology) was diluted 1:6000 and used as a loading control. Subsequently, it was incubated with the secondary antibody. The secondary antibody used was monoclonal goat anti‐mouse immunoglobulin/biotinylated (Dako) or polyclonal goat anti‐rabbit immunoglobulin/biotinylated (Dako) diluted 1:3000. The membrane was photographed with the AE‐9300 EZ‐Capture MG (manufactured by ATTO) using the EZ West Lumi One (manufactured by ATTO). The fluorescence intensity was analyzed using CS Analyzer ver. 3.0 (ATTO).

### 2.10 Statistical analysis

Multiple groups were compared using one‐way ANOVA followed by Tukey–Kramer test. The significant difference test was performed at a risk rate of 5% or 1%, and the data were shown by mean ± SEM.

## 3 RESULTS

### 3.1 Effect of MFE intake on body weight and adipose tissue weight

**FIGURE 1:**
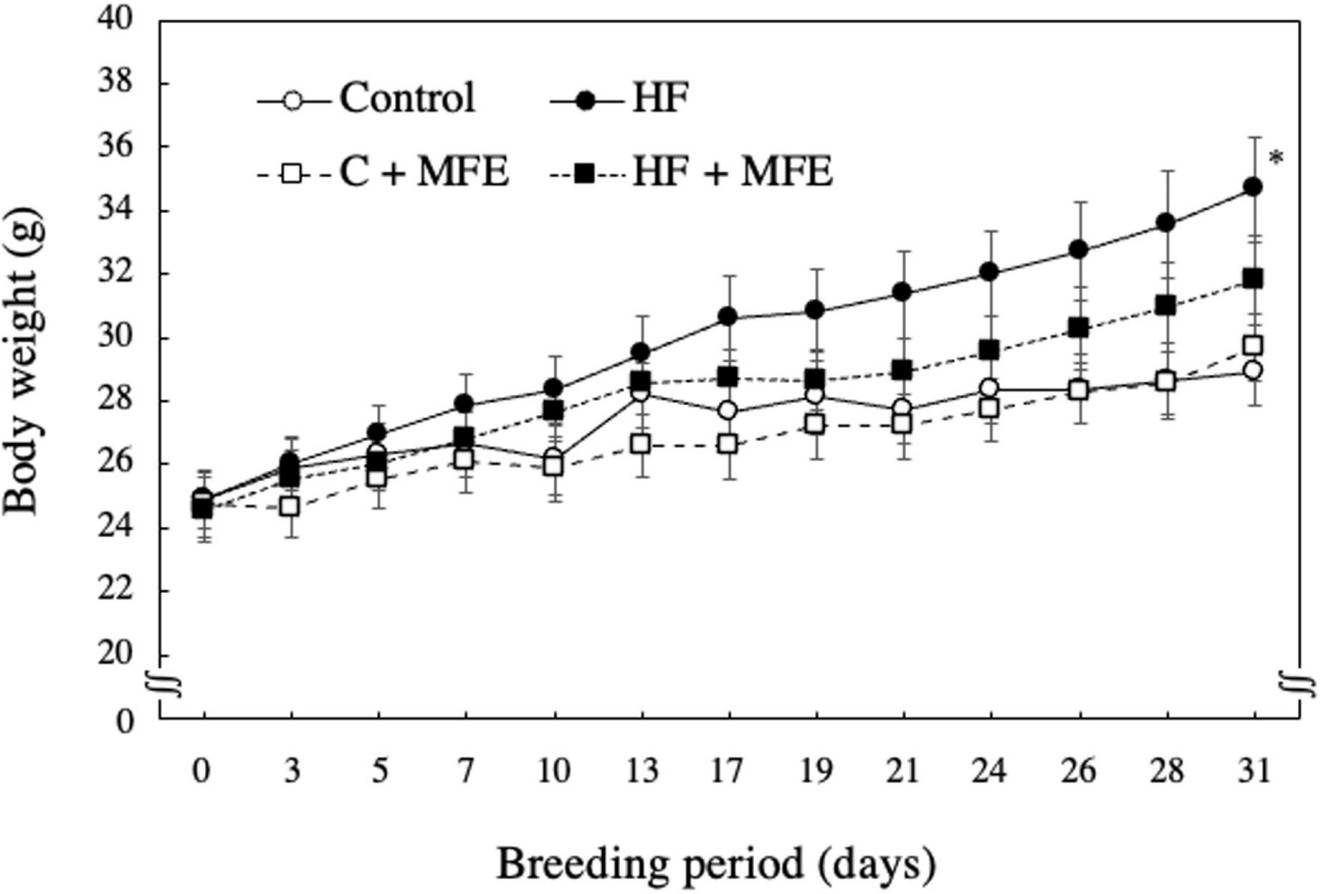
Effect of the MFE on body weight. Control; Control diet group, HF; High‐fat diet group, C + MFE; control + 0.24% MFE diet group; HF + MFE; High‐fat + 0.24% MFE diet group. Values are means ± SEM of six mice. Groups were compared using one‐way ANOVA followed by Tukey–Kramer test. *p < .05 versus control.

**FIGURE 2:**
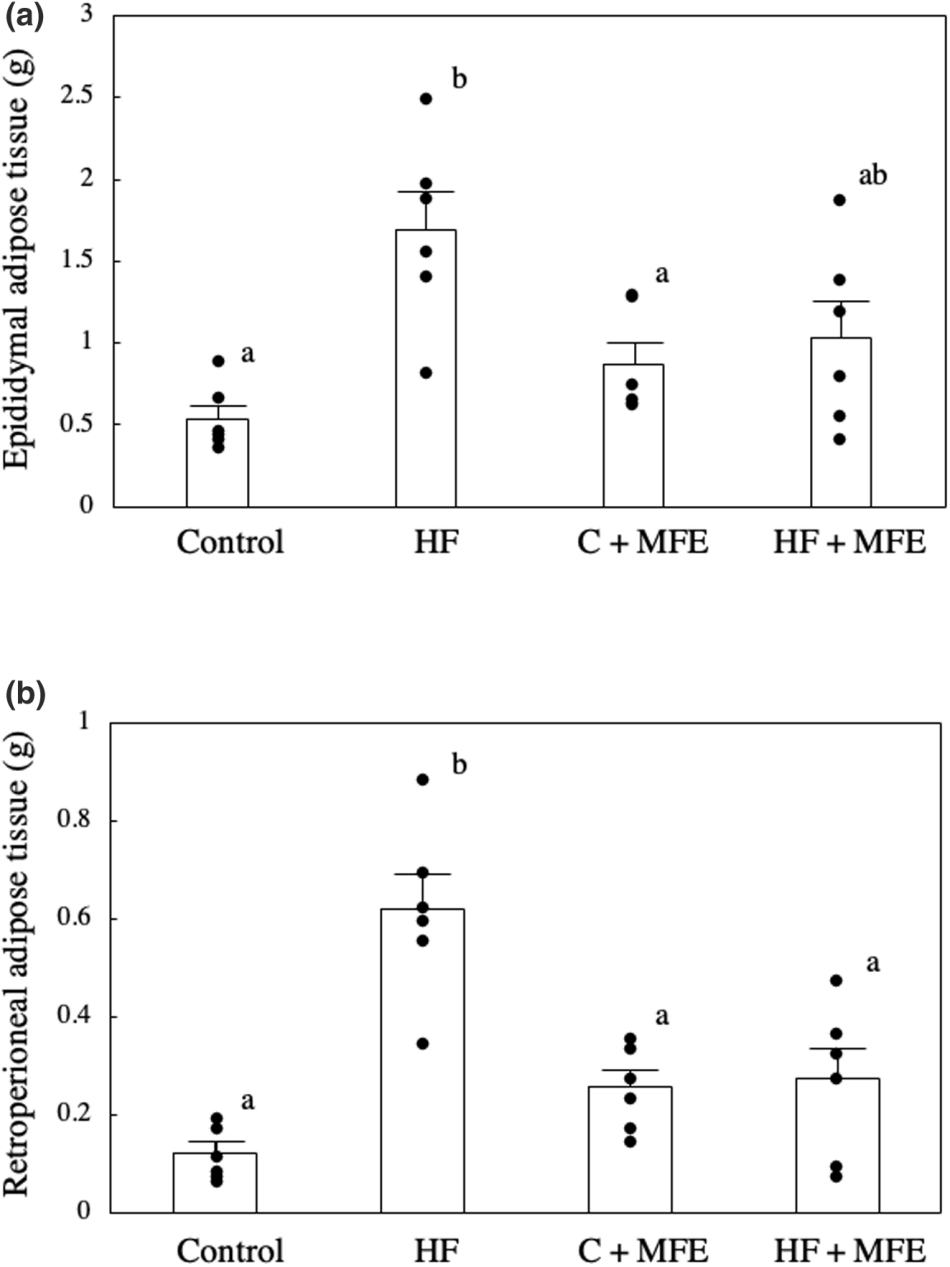
Effect of MFE on epididymal fat weight (A) and retroabdominal fat weight (B). Control: Control diet group; HF: High‐fat diet group; C + MFE: Control diet + 0.24% MFE diet group; HF + MFE; High‐fat + 0.24% MFE diet group, Values are means ± SEM of six mice. Groups were compared using one‐way ANOVA followed by Tukey–Kramer test. Values with different letters are significantly different (*p* < .05).

The body weight of mice fed an HF diet was significantly increased compared to mice fed a control diet. However, the body weight of the mice fed the HF + 0.24% MFE diet was significantly lower than the body weight of the mice fed the HF diet (Figure 1). We performed regression analysis using the data on the body weight of mice and obtained the *p*‐value and *F*‐value for all groups (*p*‐value = .027 and *F*‐value = 3.76). Next, the weight of epididymal adipose tissue increased significantly with high‐fat diet intake but tended to decrease with HF + 0.24% MFE diet intake (*p*‐value = .0017 and *F*‐value = 7.27; Figure 2a). In addition, the retroperitoneal fat weight, which was significantly increased by feeding an HF diet, was significantly decreased by ingesting MFE (*p*‐value = .000013 and *F*‐value = 16.46; Figure 2b). On the other hand, when comparing the weights of epididymal adipose tissue and retroperitoneal adipose tissue between the control diet and the control + 0.24% MFE diet, no significant difference was observed. However, there was a tendency of increased weight observed with the 0.24% MFE diet. These results differ from those observed in the case of the HF diet. These findings suggest that the addition of MFE may have beneficial effects in reducing body weight and adipose tissue weight in mice on an HF diet.

### 3.2 Effect of MFE on morphological changes in adipose tissue and liver of mice

**FIGURE 3:**
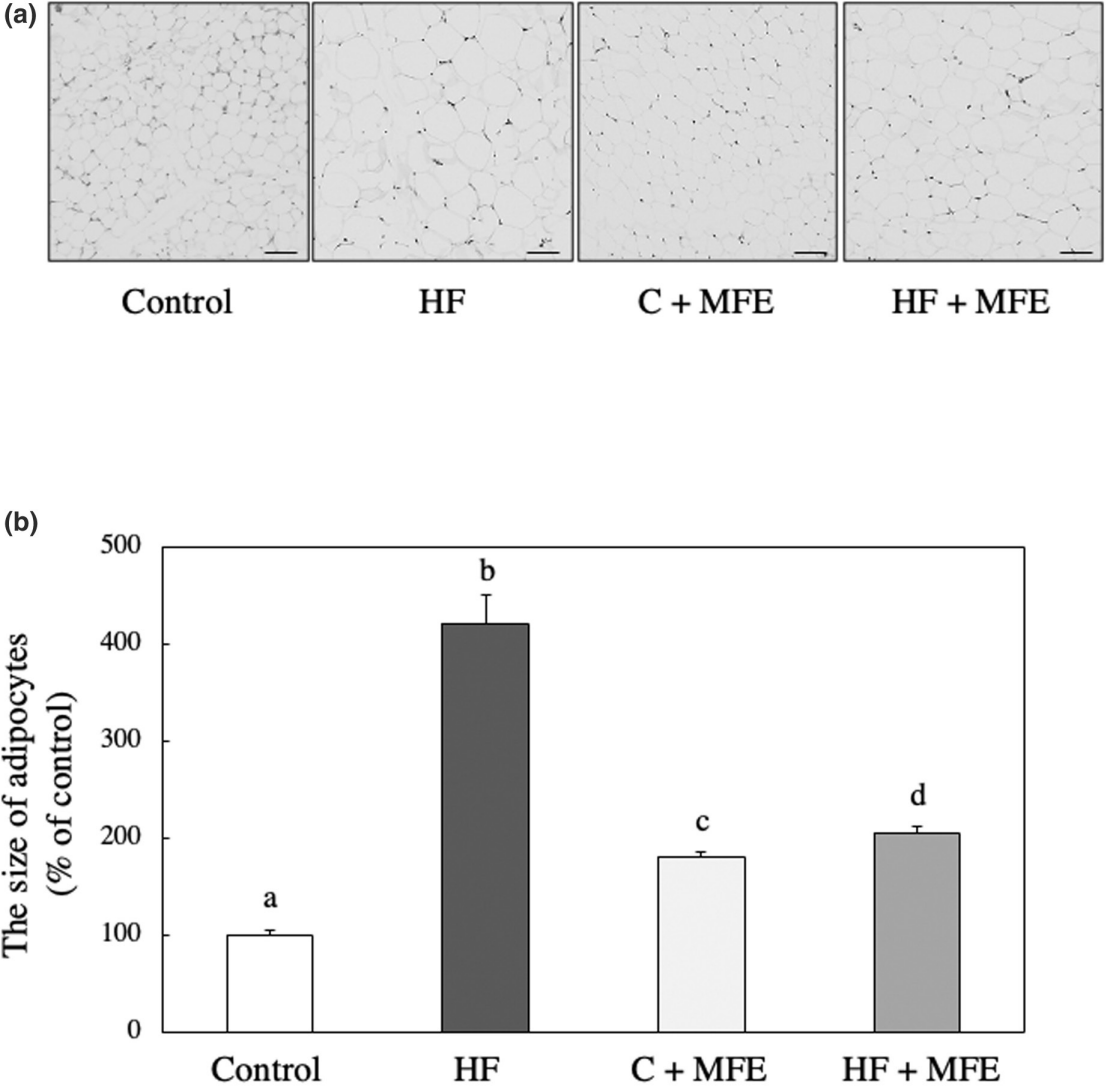
Effect of MFE on (A) morphological changes and (B) the size of adipocytes in the adipose tissue of mice. (A) Retroabdominal adipose tissue specimens were stained with H&E. (B) The size of 30 adipocytes in retroabdominal adipose tissue specimens stained with H&E was quantified using ImageJ. Control: Control diet group; HF: High‐fat diet group; Control + MFE: Control diet group + 0.24% MFE; HF + MFE: High‐fat diet group + 0.24% MFE. Values are means ± SEM of six mice. Groups were compared using one‐way ANOVA followed by Tukey–Kramer test. Values with different letters are significantly different (*p* < .05). Scale in (a) indicates 100 μm.

**FIGURE 4:**
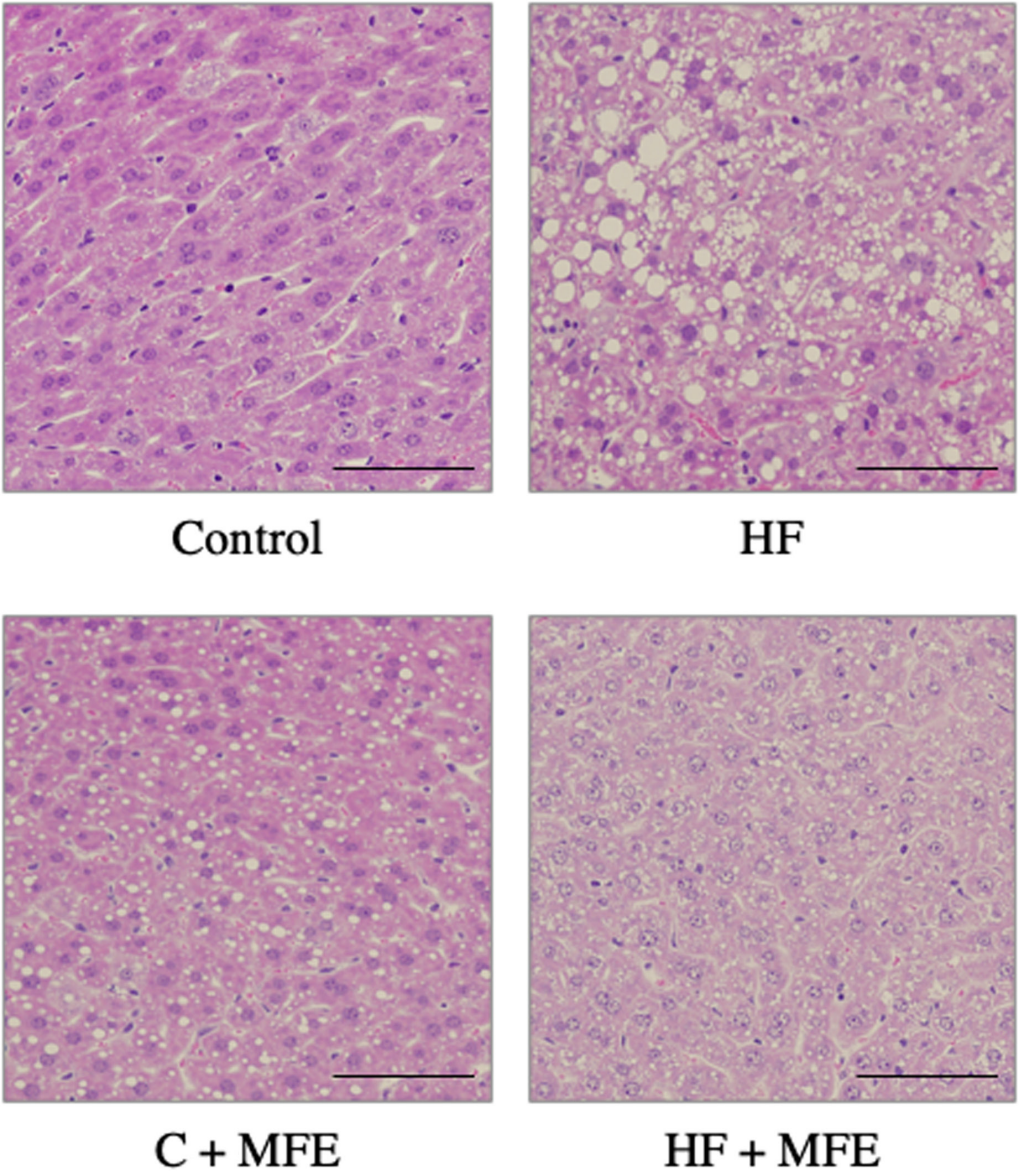
Effects of MFE on morphological changes in mouse livers. Liver specimens were stained with H&E staining. Control: Control diet group; HF: High‐fat diet group; Control + MFE: Control diet group + 0.24% MFE; HF + MFE: High‐fat diet group + 0.24% MFE. Original magnification: 20×. Scale indicates 50 μm.

The sizes of 30 adipocytes in retroabdominal adipose tissue specimens stained with H&E of each group were quantified using ImageJ. The size of adipocytes in HF diet mice was significantly larger compared to control diet mice. However, the adipocyte size was significantly smaller in mice on the HF + 0.24% MFE diet. On the other hand, when comparing the size of adipocytes between the control + 0.24% MFE diet and the control diet, a phenomenon was observed where the former showed a slightly larger size, contrary to the case with the HF diet (*p*‐value = .0000 and *F*‐value = 5018.57; Figure 3b). We also performed H&E staining on the liver specimens. In the HF group, lipid droplets accumulated in hepatocytes, indicating the development of hepatic steatosis. However, this was not observed in HF + 0.24% MFE‐fed mice (Figure 4). The findings suggest that the addition of 0.24% MFE to the HF diet resulted in a significant decrease in the size of adipocytes, indicating potential antiobesity effects. Moreover, MFE supplementation prevented the development of hepatic steatosis, highlighting its potential protective role against liver damage associated with an HF diet.

### 3.3 Effect of MFE treatment on fat formation of 3T3‐L1 preadipocytes

**FIGURE 5:**
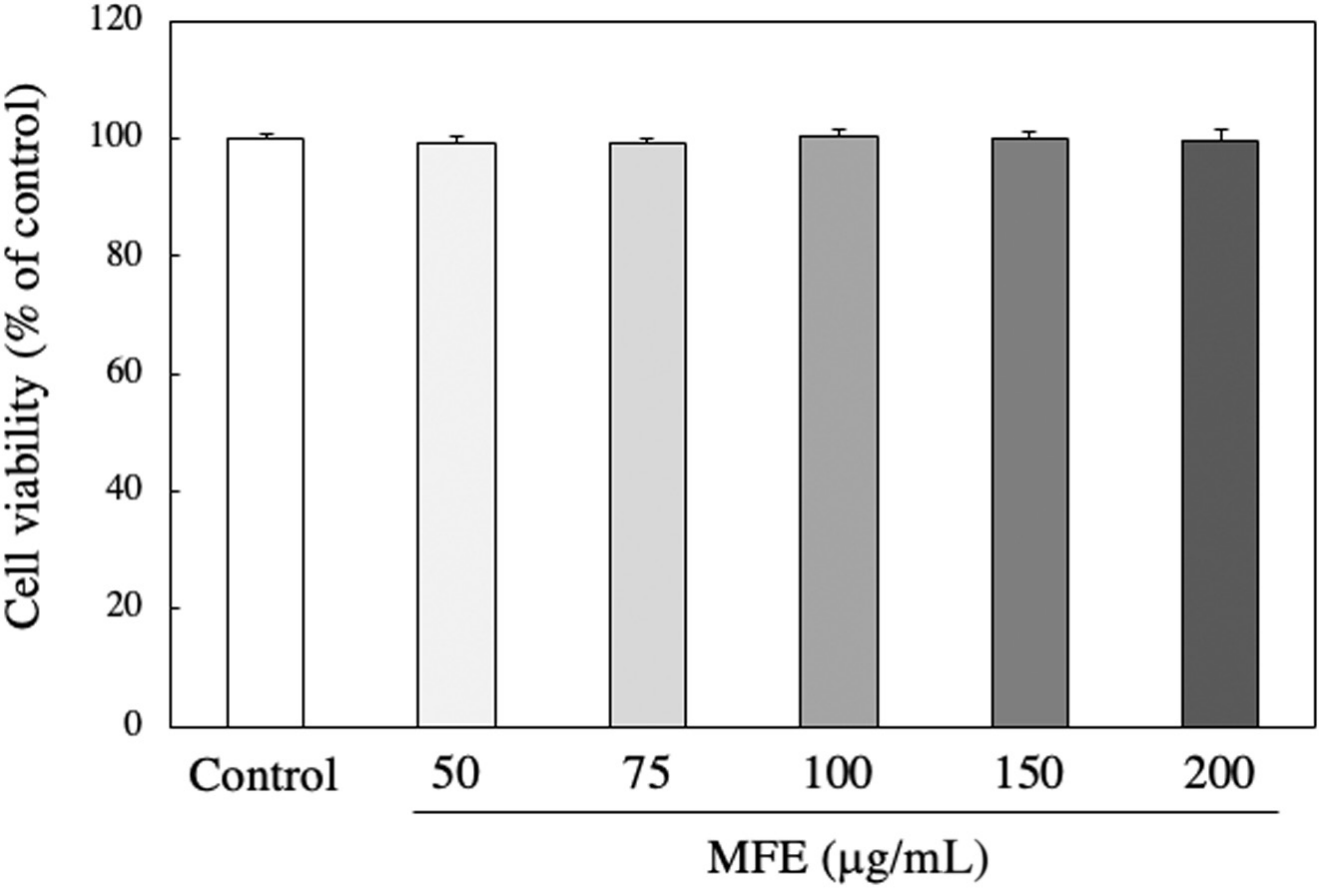
Effect of MFE on the viability of 3T3‐L1 preadipocytes. 3T3‐L1 preadipocytes were cultured with 50~200 μg/mL MFE for 24 h. The cell viability was measured using the neutral red reagent. Groups were compared using one‐way ANOVA followed by Tukey–Kramer test. The results represent the means ± SD of six experiments.

**FIGURE 6:**
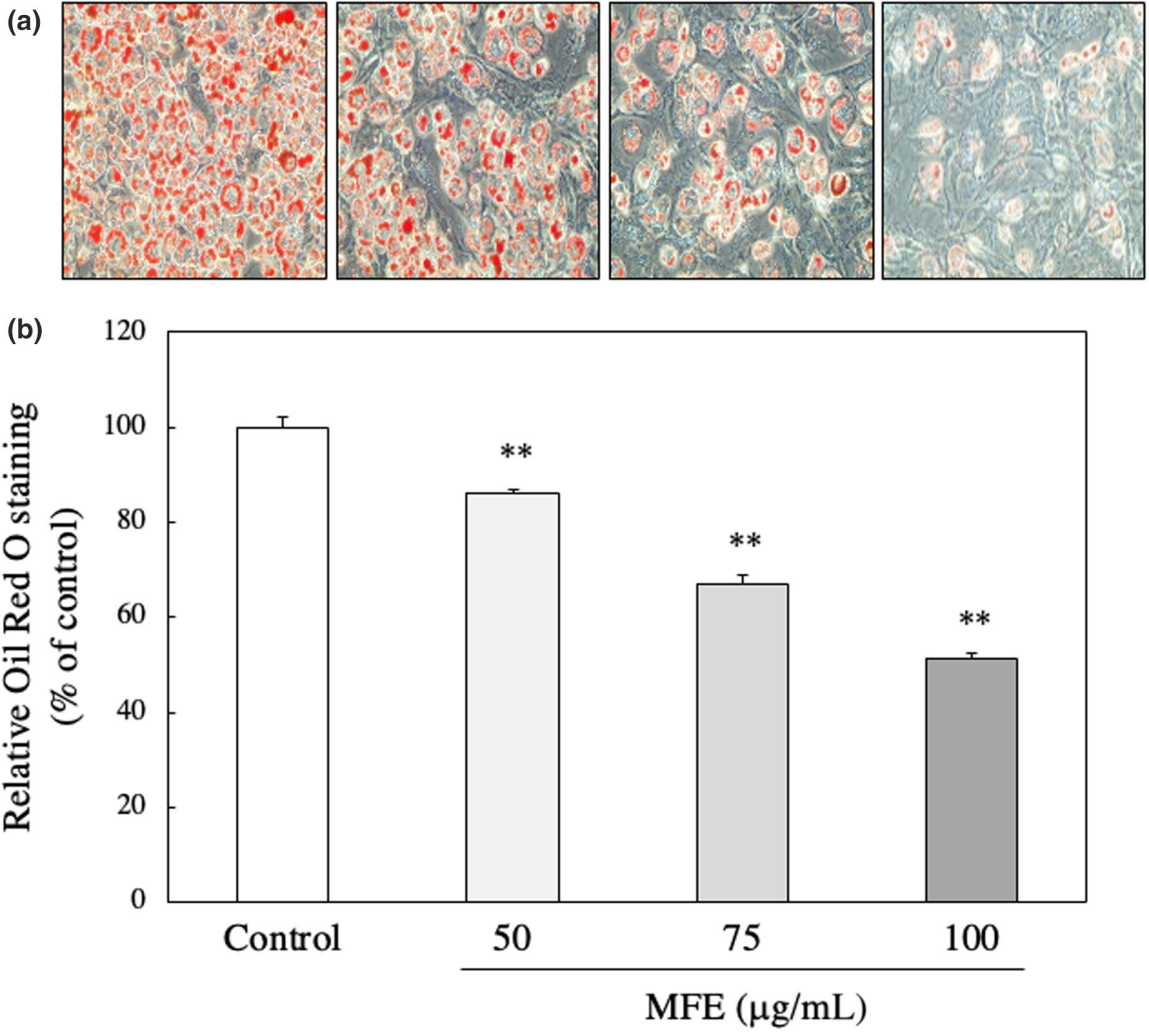
Effect of MFE on TG accumulation of 3T3‐L1 preadipocytes. Intracellular TG was stained by the Oil Red O staining method. Oil Red O staining was performed 8 days after the initiation of differentiation. (a) Representative photos of Oil Red O staining and (b) quantitative analyses of 3T3‐L1 preadipocytes treated with 0, 50, 75, or 100 μg/mL MFE. Oil Red O was extracted, and the absorbance of the extract was measured at a wavelength of 520 nm using a spectrophotometer. Groups were compared using one‐way ANOVA followed by Tukey–Kramer test. Values are means ± SD (*n* = 4). **p < .01 versus control.

**FIGURE 7:**
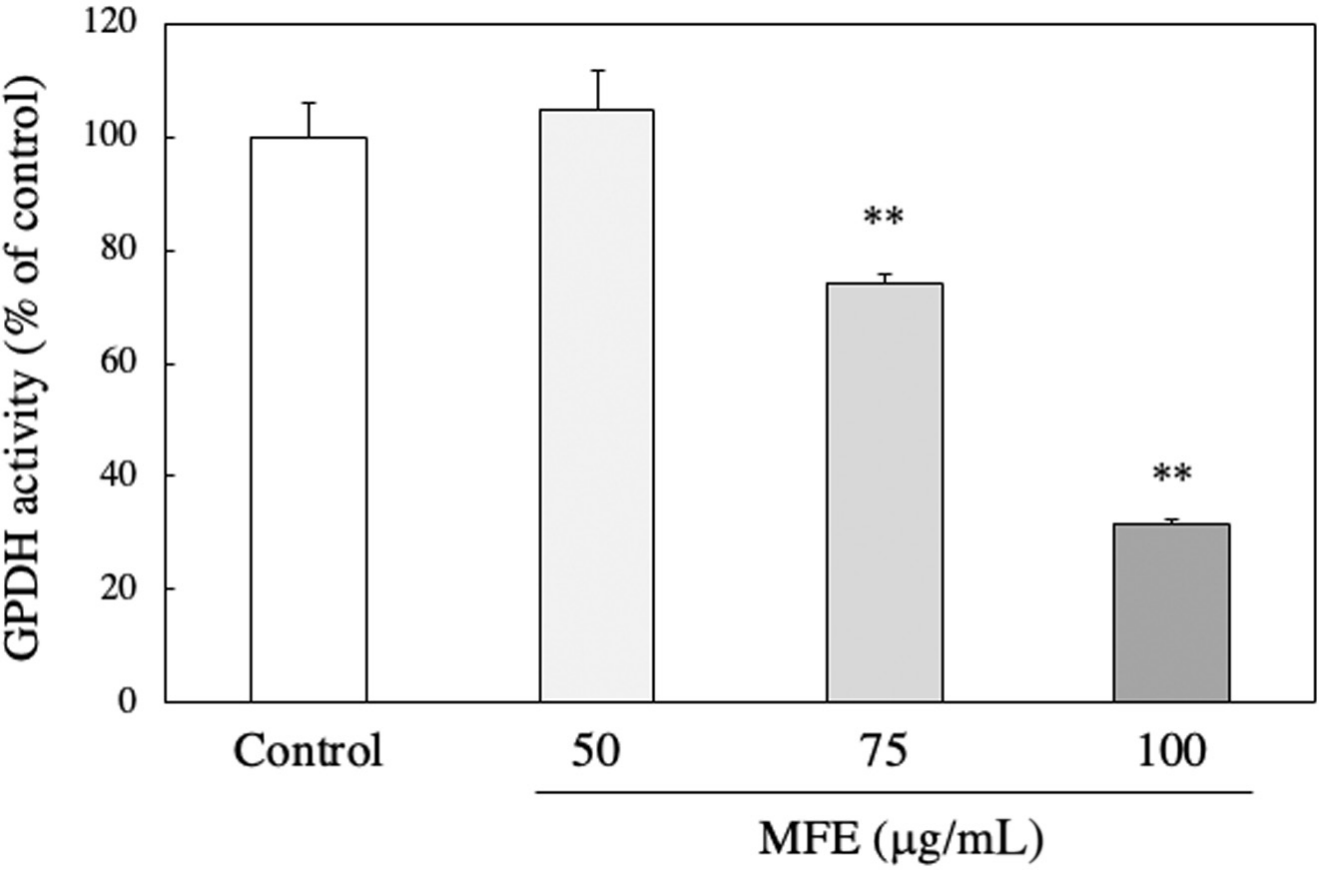
Effect of MFE on GPDH activity in 3T3‐L1 preadipocytes. The activity of GPDH was calculated using the extinction coefficient of NADH, 6.22 mM^−1^ cm^−1^, and was calculated based on the decrease in NADH every 3 min. Enzyme activity is expressed as a value relative to the control (100%). Groups were compared using one‐way ANOVA followed by Tukey–Kramer test. Values are means ± SD (*n* = 4). **p < .01 versus control.

**FIGURE 8:**
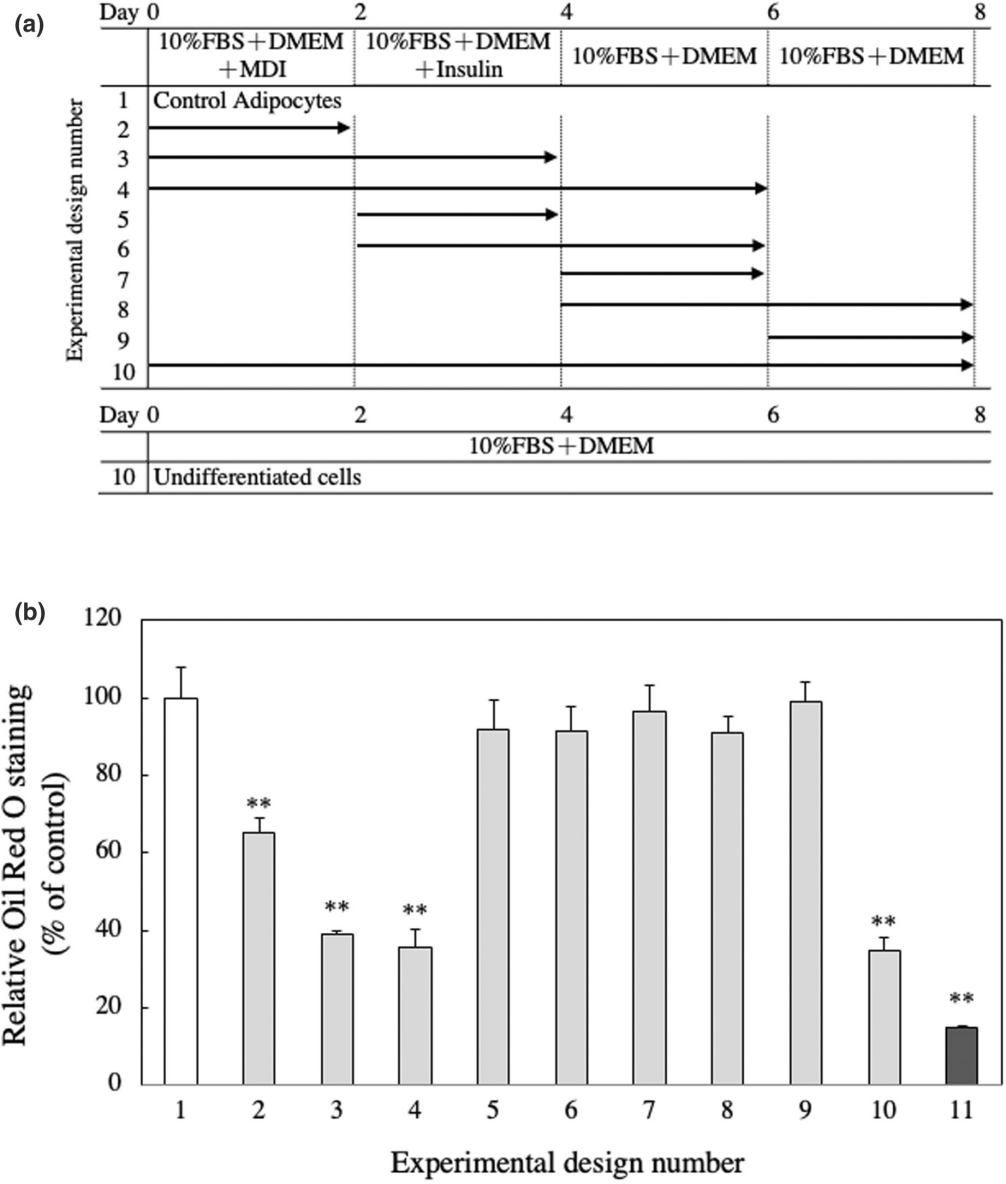
Effect of the additional period of MFE on fat accumulation in 3T3‐L1 preadipocytes. (a) The schema shows the addition of MFE during the induction of differentiation. Oil Red O staining was performed 8 days after the initiation of differentiation. (b) Oil Red O was extracted, and the absorbance of the extract was measured at a wavelength of 520 nm using a spectrophotometer. Groups were compared using one‐way ANOVA followed by Tukey–Kramer test. Values are means ± SD (*n* = 4). **p < .01 versus control.

In vivo experiments revealed that MFE significantly reduced adipose tissue weight and had an antiobesity effect, so a detailed mechanism was investigated using 3T3‐L1 cells. First, whether MFE has cytotoxicity against 3T3‐L1 cells was examined using the neutral red method. As shown in Figure 5, it was found that MFE did not affect the cell viability at a concentration of 200 μg/mL or less. Therefore, in the subsequent experiments, MFE with a concentration of 100 μg/mL or less, which was confirmed to be noncytotoxic, was used. Then, the intracellular TG accumulation amount was measured using the Oil Red staining method. The amount of TG accumulated was significantly reduced depending on the concentration of MFE added (*p*‐value = .0000 and *F*‐value = 218.08; Figure 6). In order to further confirm the suppression of TG accumulation by MFE, the effect of MFE on the activity of GPDH, the rate‐limiting enzyme of TG biosynthesis, was investigated. As shown in Figure 7, GPDH activity also decreased in a dose‐dependent manner (*p*‐value = .0000 and *F*‐value = 53.57). In order to clarify at which stage of differentiation MFE acts, it was added at various stages of differentiation and its effect on fat accumulation on the 8th day of differentiation was investigated. As shown in Figure 8, it was confirmed that exposure to MFE on day 0 reduced intracellular TG accumulation, but exposure to MFE after 2 days did not suppress TG accumulation. The results indicated that MFE reduced intracellular TG accumulation, likely through the inhibition of GPDH activity, and its effects were pronounced when administered within 2 days, early during the differentiation process.

### 3.4 Effect of MFE treatment on the expression of transcription factors involved in adipocyte differentiation of 3T3‐L1 preadipocytes

**FIGURE 9:**
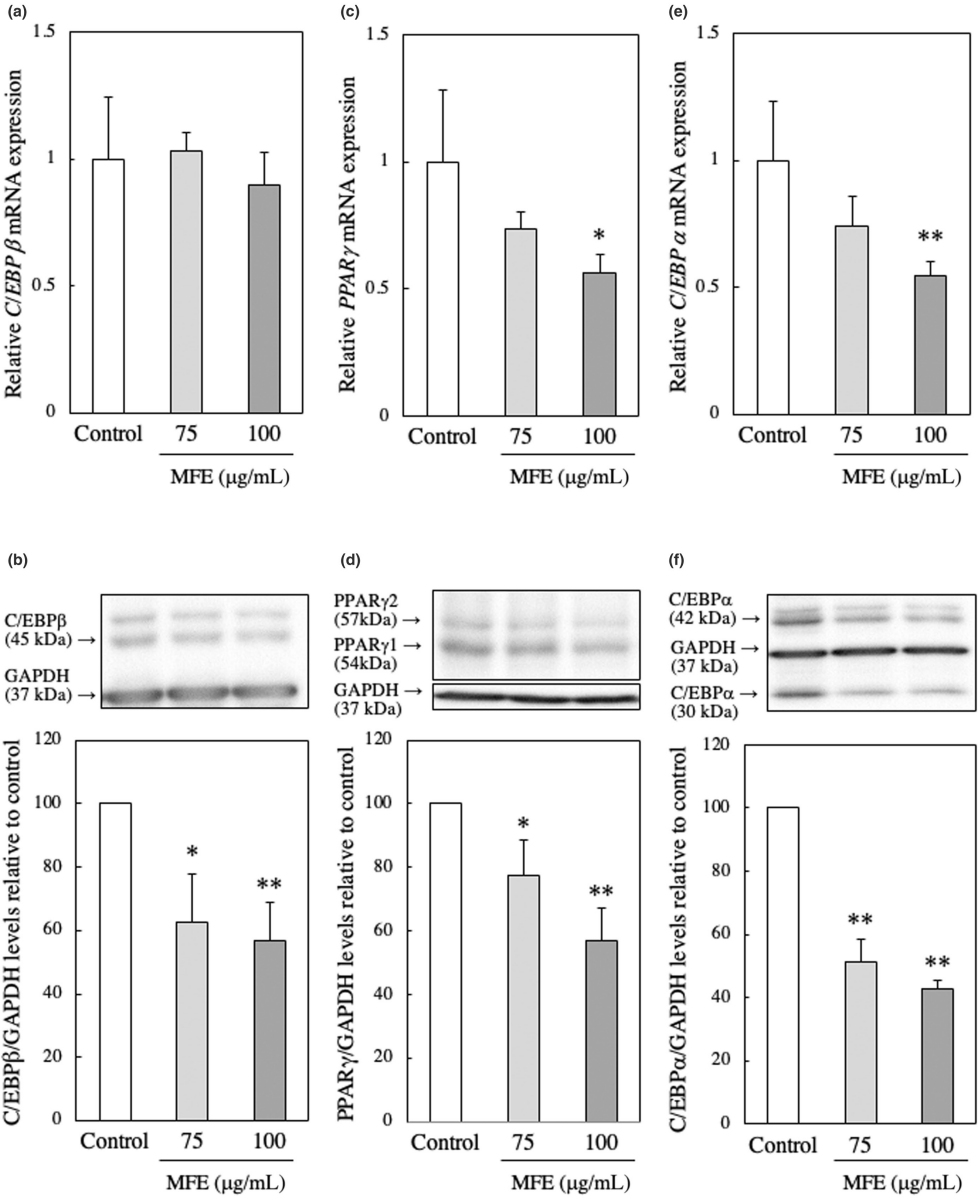
Effect of MFE on (a, c, e) the gene expression and (b, d, f) protein levels of C/EBPβ, PPARγ, and C/EBPα. mRNA expression was analyzed by real‐time PCR 48 h after induction of 3T3‐L1 preadipocyte differentiation. Values are means ± SD (*n* = 3). Protein levels were measured by Western blotting 48 h after the induction of 3T3‐L1 preadipocyte differentiation. Groups were compared using one‐way ANOVA followed by Tukey–Kramer test. Values are means ± SD (*n* = 3). **p < .01, *p < .05 versus control.

The aim of the study was to examine the impact of MFE on the expression of various transcription factor genes and proteins involved in adipocyte differentiation in 3T3‐L1 preadipocytes. It is known that during the early stages of adipocyte differentiation, C/EBPβ is expressed. Thus, we investigated the effect of MFE on the expression of C/EBPβ 48 h after initiating the differentiation of 3T3‐L1 preadipocytes. In Figure 9a,b, it can be seen that the gene expression level of C/EBPβ remained unchanged even with the addition of MFE (*p*‐value = .513 and *F*‐value = 0.72), but the protein expression level significantly decreased (*p*‐value = .007 and *F*‐value = 13.10).

In adipocytes, increased expression of C/EBPβ leads to the induction of PPARγ and C/EBPα, which are master regulators of differentiation. Therefore, we also examined the effect of MFE on the expression of PPARγ and C/EBPα 48 h after initiating the differentiation of 3T3‐L1 precursor adipocytes. The results, as shown in Figure 9c–f, demonstrated that the addition of MFE reduced both the gene expression level and protein expression level of PPARγ (gene expression level: *p*‐value = .0075 and *F*‐value = 8.86, protein expression level: *p*‐value = .0029 and *F*‐value = 17.97) and C/EBPα (gene expression level: *p*‐value = .018 and *F*‐value = 6.46, protein expression level: *p*‐value = .0000 and *F*‐value = 148.21). These findings suggest that MFE inhibits the expression of C/EBPβ through posttranslational modifications (PTMs), resulting in the transcriptional suppression of PPARγ and C/EBPα. The sequential suppression of these key transcription factors may contribute to the observed inhibition of differentiation in 3T3‐L1 cells when treated with MFE.

### 3.5 Effect of MFE treatment on posttranslational modification (PTM) of C/EBPβ protein in 3T3‐L1 preadipocytes

**FIGURE 10:**
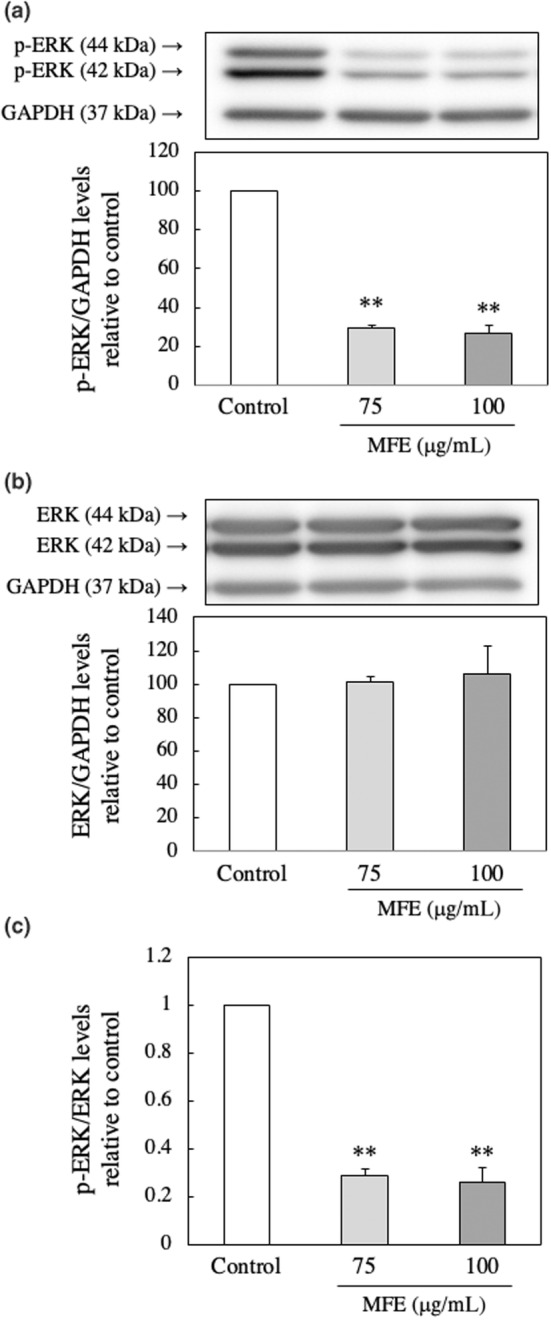
Effect of MFE on the phosphorylation of ERK. p‐ERK (a), ERK (b), and p‐ERK/ERK (c) protein levels were measured by Western blotting 1 h after the induction of 3T3‐L1 preadipocytes differentiation. Groups were compared using one‐way ANOVA followed by Tukey–Kramer test. Values are means ± SD (*n* = 3). **p < .01 versus control.

**FIGURE 11:**
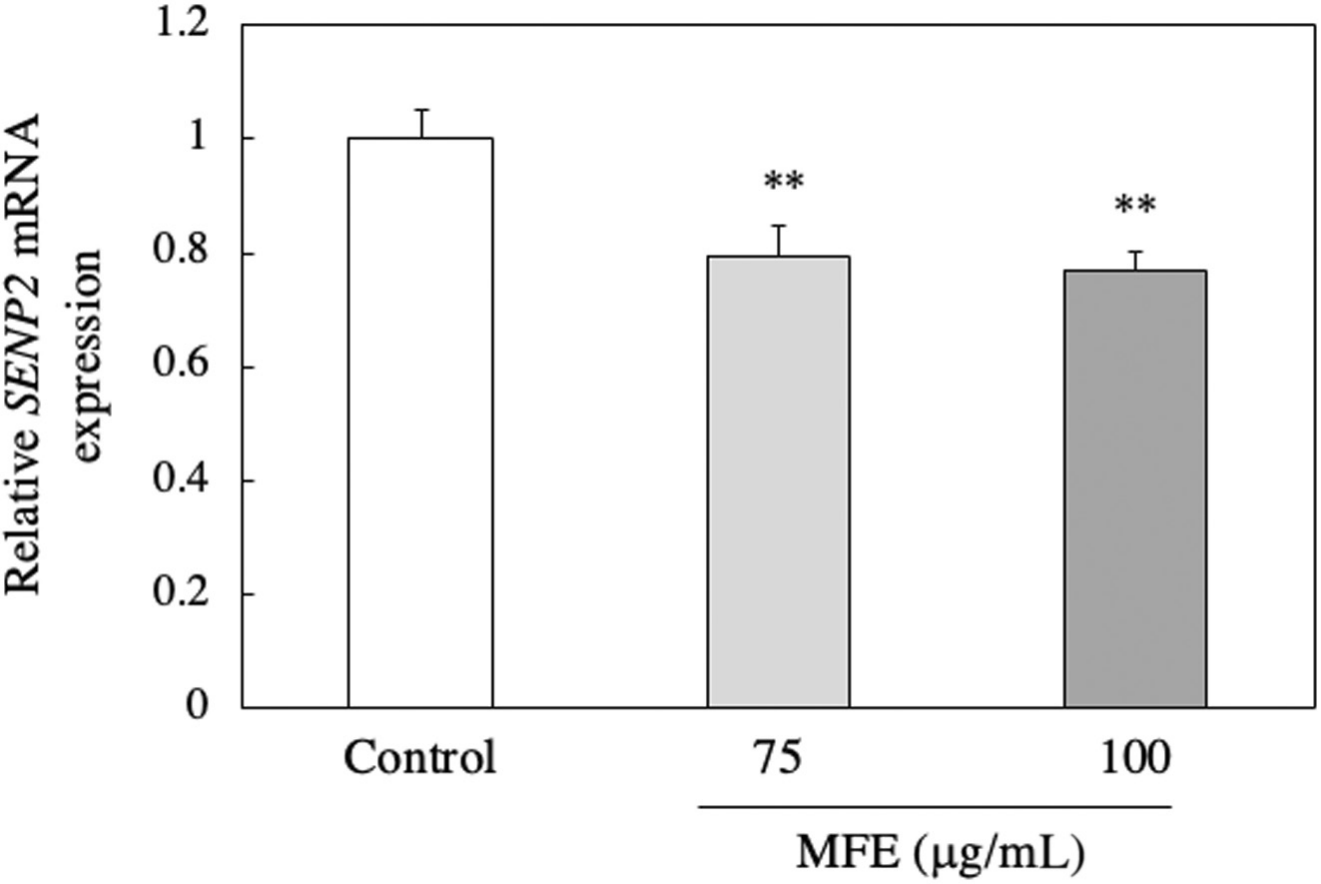
Effect of MFE on the expression of *SENP2* mRNA. *SENP2* mRNA expression was analyzed by real‐time PCR 4 h after induction of 3T3‐L1 preadipocyte differentiation. Groups were compared using one‐way ANOVA followed by Tukey–Kramer test. Values are means ± SD (*n* = 3). **p < .01 versus control.

MFE did not affect the C/EBPβ gene expression level of 3T3‐L1 preadipocytes and suppressed the protein expression level. This suggests that MFE may be involved in PTM of the C/EBPβ protein. Therefore, the effect of MFE treatment on the factors related to PTM of C/EBPβ protein was investigated. We examined the involvement of ERK, which is one of the members of the MAPK family. ERK is active by being phosphorylated and is involved in the expression of C/EBPβ protein (McCubrey et al., 2007). One hour after the induction of differentiation of 3T3‐L1 precursor adipocytes, MFE suppressed the protein expression level of phosphorylated ERK (p‐ERK; *p*‐value = .0000 and *F*‐value = 739.24; Figure 10a), but did not affect the protein expression level of ERK (*p*‐value = .762 and *F*‐value = 0.29; Figure 10b). From this, it was clarified that MFE suppresses the phosphorylation of ERK (*p*‐value = .0000 and *F*‐value = 224.63; Figure 10c). Furthermore, Chung et al. reported that the stability of C/EBPβ protein is increased by deSUMOylation (Chung et al., 2010). Therefore, we examined the involvement of SUMOylation (small ubiquitin‐related modifier), one of PTMs. We assayed the effect of MFE on the expression of sentrin/SUMO‐specific protease 2 (SENP2), which is a deSUMOase. As shown in Figure 11, MFE suppressed the SENP2 gene expression level 4 h after the induction of differentiation of 3T3‐L1 preadipocytes (*p*‐value = .0001 and *F*‐value = 29.71). The findings suggest that MFE influence the PTM of the C/EBPβ protein. It was observed that MFE suppresses the phosphorylation of ERK and downregulation of the gene expression level of SENP2, potentially affecting the stability and activity of the C/EBPβ protein. These findings provide insights into the molecular mechanisms underlying the inhibitory effects of MFE on adipocyte differentiation.

## 4 DISCUSSION

In this study, we demonstrated that the treatment of MFE suppresses the expression of transcription factors that involves the early stage of differentiation of 3T3‐L1 preadipocytes and reduces TG synthesis ability and TG accumulation. An interesting finding in this study was the clarification that MFE decreases the expression of C/EBPβ through post‐translation modifications (PTMs), followed by the transcriptional suppression of PPAR𝛾 and C/EBPα.

Exposure to MFE for 8 days from the start of differentiation induction suppressed TG accumulation and TG synthesis ability in preadipocytes. Therefore, in order to investigate at which stage of differentiation MFE affects, MFE was added to various stages of differentiation, and the effect on fat accumulation was investigated. Then, we revealed that MFE exposure from the start to the second day was necessary. These results clarified that MFE suppresses the differentiation by some influence in the early stage of the differentiation of 3T3‐L1 preadipocytes.

Therefore, the gene expression levels of transcriptional regulators (C/EBPβ, PPARγ, and C/EBPα) involved at an early stage of adipocyte differentiation were examined by the qRT‐PCR method and the protein level by the Western blotting method. In adipocytes, the expression of C/EBPβ and C/EBPδ is induced in the early stage of differentiation, and these induce the expression of PPARγ and C/EBPα, which are master regulators of adipocyte differentiation (Cao et al., 1991; Wu et al., 1996). PPARγ and C/EBPα induce the expression of adipocyte‐specific factors while activating each other's expression (MacDougald & Lane, 1995) and promoting adipocyte differentiation. First, we investigated the effect of MFE on the expression of C/EBPβ mRNA level and found that the gene expression level of C/EBPβ 48 h after the induction of differentiation did not change even when MFE was added (Figure 9a). On the other hand, the protein level of C/EBPβ decreased significantly depending on the concentration of MFE added (Figure 9b). Next, the effect of MFE on the expression of PPARγ was investigated, and found that MFE significantly reduced both the gene expression level (Figure 9c) and protein level (Figure 9d) of PPARγ at 48 h after the induction of differentiation. Furthermore, MFE also significantly reduced both gene and protein levels of C/EBPα at 48 h after the induction of differentiation. These results indicated that MFE suppresses the expression of PPARγ and C/EBPα through the PTMs of C/EBPβ protein.

PTMs, including phosphorylation, acetylation, methylation, ubiquitination, SUMOylation, glycosylation, *etc*., profoundly affect the function of proteins and play important roles in many biological processes (Tuk et al., 2019). When C/EBPβ is phosphorylated, it forms a dimer via the leucine zipper domain at the C‐terminal (Guo et al., 2015), making it less susceptible to proteolysis by calpain and stabilizing (Zhang et al., 2012). Furthermore, the C/EBPβ protein acquires DNA‐binding ability and promotes transcriptional activity (Guo et al., 2015). Furthermore, SUMOylation is a modification in which the ubiquitin‐like protein SUMO covalently binds to a target protein to control its function. It is involved in the intracellular localization of proteins, nuclear/cytoplasmic transport, stability, and interactions (Edward & Yeh, 2009). The C/EBPβ protein is degraded by the proteasome by binding SUMO to Lys133 (Liu et al., 2013). Therefore, in order to investigate the mechanism that regulates PTMs of C/EBPβ protein by MFE, we focused on phosphorylation and SUMOylation.

In C/EBPβ, Thr188 is phosphorylated mainly by the MAPK family ERK and p38 (Pulido‐Salgado et al., 2015). Here, we investigated the effect of MFE on the protein levels of intracellular ERK and p‐ERK. As shown in Figure 10, MFE did not affect the protein level of ERK 1 h after the induction of differentiation (Figure 10b) but significantly reduced the protein level of p‐ERK in a dose‐dependent manner (Figure 10a). From these results, it was clarified that MFE suppresses the phosphorylation of ERK protein. On the other hand, SENP2, an enzyme that promotes deSUMOylation, increases the stability of C/EBPβ protein (Chung et al., 2010). Therefore, the effect of MFE on the gene expression level of the deSUMOase SENP2 was investigated. We found that MFE significantly reduced the SENP2 gene expression level 4 h after the induction of differentiation in a concentration‐dependent manner (Figure 11). These results suggest that MFE also regulates SUMOylation of C/EBPβ protein by suppressing SENP2 gene expression. There is a report that the interaction between PTMs modulates the function of the target protein. Gregoire et al. found that phosphorylation is necessary for subsequent SUMOylation to inhibit the transcriptional and myogenic activities of MEF2 (Gregoire et al., 2006). This suggests that the regulation of C/EBPβ expression requires coordinated actions of phosphorylation and SUMOylation. However, this issue needs further research.

In the in vivo experiment, we confirmed that MFE significantly suppressed the increase in body weight in the in vivo obesity model HF diet mice. We also observed by histological analysis that the liver of the HF group was fatty liver. In the HF + MFE group, on the other hand, the occurrence of fatty liver was significantly suppressed (Figure 4). These results suggest that MFE inhibits hepatic steatosis induced by HF diet. MFE also suppressed the increase in the weights of epididymal and retroperitoneal adipose tissue caused by the HD diet. However, there was a tendency of increased weight observed in those adipose tissues with the control + 0.24% MFE diet compared with the control diet. The tendency for adipocyte tissue weight to increase with the addition of 0.24% MFE in the control diet, unlike the case with the HF diet, is an interesting finding. This trend was further pronounced in terms of adipocyte size within the adipose tissue. Significant differences were observed in the size of adipocytes between the control diet and the 0.24% MFE diet. The reason for this phenomenon is not yet clear at this stage. When examining the feed efficiency during the experiment, the following values were obtained: control diet: 1.87%; control + 0.24% MFE diet: 2.80%; HF diet: 6.39%; and HF + 0.24% MFE diet: 5.15%. These results indicate that the addition of MFE reduced feed efficiency in the HF diet but increased it in the control diet. This suggests that MFE may have effects other than its antiobesity properties. This is a highly intriguing phenomenon that requires further research in the future.

In conclusion, MFE has an antiobesity effect by controlling the phosphorylation and SUMOylation of C/EBPβ protein in the early stage of differentiation of 3T3‐L1 preadipocytes. MFE has the potential to be a new therapeutic agent effective in preventing obesity.

## AUTHOR CONTRIBUTIONS

**Touko Nakano:** Formal analysis (lead); investigation (lead); methodology (lead); writing – original draft (lead). **Yutaro Sasaki:** Formal analysis (equal); investigation (equal); methodology (equal). **Toshio Norikura:** Formal analysis (equal); investigation (equal); methodology (equal). **Yusuke Hosokawa:** Formal analysis (equal); investigation (equal); methodology (equal). **Mayu Kasano:** Formal analysis (equal); investigation (equal); methodology (equal). **Isao Matsui‐Yuasa:** Formal analysis (equal); investigation (equal); methodology (equal); writing – original draft (lead). **Xuedan Huang:** Resources (lead). **Yoshinori Kobayashi:** Resources (lead). **Akiko Kojima‐Yuasa:** Formal analysis (equal); funding acquisition (lead); investigation (equal); methodology (equal); project administration (lead); supervision (lead); writing – original draft (lead).

## FUNDING INFORMATION

This work was supported by JSPS KAKENHI (grant number 15K00832).

## CONFLICT OF INTEREST STATEMENT

The authors declare that they do not have any conflict of interest.

## ETHICS STATEMENT

Animal experiments were conducted in accordance with the Osaka City University Animal Experiment Committee Regulations (Permit No. S0056).

## Data Availability

The data that support the findings of this study are available on request from the corresponding author upon reasonable request.
